# Association between antibiotic use during early life and early‐onset colorectal cancer risk overall and according to polygenic risk and *FUT2* genotypes

**DOI:** 10.1002/ijc.34648

**Published:** 2023-07-28

**Authors:** Fangyuan Jiang, Daniel Boakye, Jing Sun, Lijuan Wang, Lili Yu, Xuan Zhou, Jianhui Zhao, Zilong Bian, Peige Song, Yazhou He, Yingshuang Zhu, Jie Chen, Shuai Yuan, Mingyang Song, Susanna C. Larsson, Edward L Giovannucci, Evropi Theodoratou, Kefeng Ding, Xue Li

**Affiliations:** ^1^ Colorectal Surgery and Oncology, Key Laboratory of Cancer Prevention and Intervention, Ministry of Education, The Second Affiliated Hospital and School of Public Health Zhejiang University School of Medicine Hangzhou China; ^2^ Department of Big Data in Health Science, School of Public Health and The Second Affiliated Hospital Zhejiang University School of Medicine Hangzhou China; ^3^ Department of Life Sciences PMI Global Studio Limited London UK; ^4^ Centre for Global Health Usher Institute, The University of Edinburgh Edinburgh UK; ^5^ West China School of Public Health and West China Fourth Hospital Sichuan University Chengdu China; ^6^ Unit of Cardiovascular and Nutritional Epidemiology Institute of Environmental Medicine, Karolinska Institute Stockholm Sweden; ^7^ Department of Epidemiology Harvard T.H. Chan School of Public Health Boston Massachusetts USA; ^8^ Department of Nutrition Harvard T.H. Chan School of Public Health Boston Massachusetts USA; ^9^ Clinical and Translational Epidemiology Unit Massachusetts General Hospital and Harvard Medical School Boston Massachusetts USA; ^10^ Division of Gastroenterology Massachusetts General Hospital Boston Massachusetts USA; ^11^ Unit of Medical Epidemiology, Department of Surgical Sciences Uppsala University Uppsala Sweden; ^12^ Cancer Research UK Edinburgh Centre, Medical Research Council Institute of Genetics and Cancer The University of Edinburgh Edinburgh UK; ^13^ Zhejiang Provincial Clinical Research Center for Cancer Cancer Center of Zhejiang University Hangzhou China

**Keywords:** antibiotic use, early‐life factors, early‐onset colorectal cancer, FUT2 gene, polygenic risk

## Abstract

Early‐onset colorectal cancer (EOCRC) has been increasing worldwide. Potential risk factors may have occurred in childhood or adolescence. We investigated the associations between early‐life factors and EOCRC risk, with a particular focus on long‐term or recurrent antibiotic use (LRAU) and its interaction with genetic factors. Data on the UK Biobank participants recruited between 2006 and 2010 and followed up to February 2022 were used. We used logistic regression to estimate adjusted odds ratios (ORs) and 95% confidence intervals (95% CIs) of the associations between LRAU during early life and EOCRC risk overall and by polygenic risk score (constructed by 127 CRC‐related genetic variants) and *Fucosyltransferase 2* (*FUT2*), a gut microbiota regulatory gene. We also assessed the associations for early‐onset colorectal adenomas, as precursor lesion of CRC, to examine the effect of LRAU during early‐life and genetic factors on colorectal carcinogenesis. A total of 113 256 participants were included in the analysis, with 165 EOCRC cases and 719 EOCRA cases. LRAU was nominally associated with increased risk of early‐onset CRC (OR = 1.48, 95% CI = 1.01‐2.17, *P* = .046) and adenomas (OR = 1.40, 95% CI = 1.17‐1.68, *P* < .001). When stratified by genetic polymorphisms of *FUT2*, LRAU appeared to confer a comparatively greater risk for early‐onset adenomas among participants with rs281377 TT genotype (OR = 1.10, 95% CI = 0.79‐1.52, *P* = .587, for CC genotype; OR = 1.75, 95% CI = 1.16‐2.64, *P* = .008, for TT genotype; *P*_interaction_ = .089). Our study suggested that LRAU during early life is associated with increased risk of early‐onset CRC and adenomas, and the association for adenomas is predominant among individuals with rs281377 TT/CT genotype. Further studies investigating how LRAU contributes together with genetic factors to modify EOCRC risk, particularly concerning the microbiome‐related pathway underlying colorectal carcinogenesis, are warranted.

## Graphical abstract

What's new?

Early‐onset colorectal cancer is on the rise worldwide, possibly driven by exposure to risk factors in childhood. Long‐term or recurrent use of antibiotics, for instance, could cause long‐lasting changes in the gut microbiota that lead to cancer susceptibility. Here, the authors investigated the association between antibiotics use and cancer risk, both overall and according to certain genetic factors. They found that long‐term or recurrent antibiotic use increased the risk of early‐onset CRC and adenomas, with a stronger effect in people with a particular variant of the gut microbiota regulatory gene, *Fucosyltransferase* 2 (FUT2).

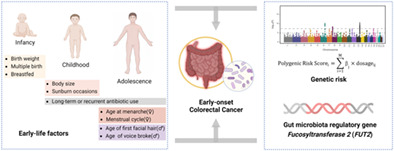

**Abbreviations**
CI: confidence interval
CRC: colorectal cancer
EOCRA: early‐onset colorectal adenomas
EOCRC: early‐onset colorectal cancer
FUT2: fucosyltransferase 2
GWAS: genome‐wide association study
LD: linkage disequilibrium
LRAU: long‐term or recurrent antibiotic use
OR: odds ratio
PRS: polygenic risk score
SNPs: single nucleotide polymorphisms

## 1 INTRODUCTION

The incidence of colorectal cancer (CRC) in populations older than 50 years is decreasing in many countries (eg, The United States, The United Kingdom), which is largely attributed to the implementation of effective population‐based screening for early detection and removal of precursors.^1^, ^2^ However, early‐onset CRC (EOCRC; diagnosed before 50 years) is increasing worldwide over the past several decades.^3^, ^4^ Given the increasing incidence of CRC among younger adults, investigating risk factors for EOCRC is important for enhancing and tailoring primary prevention in this population of special interest.

Accumulating evidence suggests that some potential risk factors for EOCRC may be operative during childhood or adolescence.^5^, ^6^ As accessibility to antibiotics increases across low and middle‐income countries, antibiotic therapy for common childhood infections or adolescent acne is increasingly widespread.^7^, ^8^ However, childhood or adolescence may be more vulnerable periods to the potential effects of overexposure.^5^, ^6^ Epidemiology studies have observed significant correlations between long‐term use of antibiotic and risk of colorectal neoplasm.^9^, ^10^, ^11^ Studies investigating potentially modifiable risk factors for EOCRC have, however, focused on lifestyle factors and exposures occurring during adolescence and/or early adulthood and found associations with several factors such as obesity^12^, ^13^ and alcohol consumption.^14^ There is thus limited evidence evaluating the effects of LRAU during early life on the risk of early‐onset colorectal neoplasm. Recent genome‐wide association studies (GWAS) have expanded the catalog of CRC risk‐related single nucleotide polymorphisms (SNPs).^15^, ^16^ Polygenic risk score (PRS) combines the effect of these SNPs and provides powerful tools for risk stratification and prediction of CRC risk, especially for EOCRC.^17^ It has been suggested that lifestyle factors could interact with PRS to affect EOCRC risk.^18^ However, to our knowledge, no previous studies have assessed such potential interactions between early‐life exposures and the genetic factors in relation to EOCRC risk.

Early‐life events could influence gut microbiota, which have been proposed to play an important role in the initiation and development of CRC.^5^, ^6^ LRAU during early life may thus cause long‐standing changes in gut microbiota, and irregularities in immunostimulatory bacterial products that can impede normal immune surveillance, increasing CRC risk.^19^ The *Fucosyltransferase 2* (*FUT2*) is a gut microbiota regulatory gene and plays an essential role in regulating gut microbiota composition.^20^, ^21^ The dysregulation of gut microbiota could be at the crossroads of the above‐mentioned risk factors contributing to EOCRC. It could thus be hypothesized that factors that impact gut microbiota content (eg, antibiotic use and genetic polymorphisms of *FUT2*) might interact together to modify EOCRC risk, but this important question has not been investigated either.

Herein, we used data from the UK Biobank (a) to investigate the associations of LRAU during early life with EOCRC risk overall and according to genetic factors, and (b) to test whether the association between LRAU and risk of EOCRC as well as adenomas differs by the genetic polymorphisms of *FUT2*.

## 2 METHODS

### 2.1 Study population

The UK Biobank is a large prospective cohort study with comprehensive health‐related phenotypic and genotypic information. It comprises over 500 000 participants, aged 40 to 69 years, recruited from across 22 centers located throughout England, Wales and Scotland between 2006 and 2010. Details of the baseline information, biological samples collection and genome‐wide genotyping have been described elsewhere.^22^

### 2.2 Early‐life factors and covariates

LRAU during early life was defined as long‐term or recurrent use of antibiotics during childhood and/or adolescence. In brief, the participants were asked whether they received long‐term or recurrent courses of antibiotics (3+ per year, eg, for acne), and the information was binary (yes/no). The UK Biobank obtained this early‐life LRAU data from 174 714 participants, utilizing a digestive health questionnaire disseminated to a subset of the cohort (~335 000 individuals) via email or through the participant website. Of this group, ~52% (176 345 participants) completed the questionnaire. In addition to LRAU during early life, we also investigated other early‐life factors, encompassing postnatal factors and age at onset of secondary sexual characteristics (more details in Table S1). Information on these early life factors was assessed via the baseline touchscreen questionnaire, verbal interview, or follow‐up questionnaire. Information on covariates, including age (continuous in years), sex (men and women), education attainment (college or university degree and above, and high school and below), was assessed in the baseline survey. History of childhood diabetes was ascertained via linkage to the main primary care computer system suppliers in England as well as the hospital inpatient data. Missing data was ~1% for education level and 20% for family history of CRC. To avoid a large amount of data loss due to missing information on a particular covariate (eg, family history of CRC), we employed multiple imputation for the missing categorical covariates.

### 2.3 Definition and ascertainment of early‐onset colorectal neoplasm

Diagnosis of EOCRC and early‐onset colorectal adenomas (EOCRA) was ascertained through linkage to national cancer registries in England, Wales and Scotland (follow‐up through to 20 February 2022) as well as inpatient medical records (follow‐up through to 31 March 2021). CRC as first or second diagnosis was identified using the International Classification of Diseases codes (ICD‐9: 153, 154.0 and 154.1; ICD‐10: C18‐C20). We defined both EOCRC and EOCRA as prevalent (before the baseline assessment) or incident (during follow‐up) diagnosis among participants aged <50 years, and this cut‐off was based on recommendations for CRC screening in high‐income countries^23^ and previous studies.^24^, ^25^, ^26^

### 2.4 Eligibility criteria

Of 502 490 participants from the UK Biobank, we excluded participants without genetic information (n = 15 249), of non‐European ancestry (n = 25 754), with other cancer diagnosis throughout lifetime (n = 127 248) and those diagnosed with EOCRC during adolescence or earlier (≤19 years, n = 2). A total of 113 256 participants with available information of LRAU were ultimately eligible for analysis (Figure S1), including 165 EOCRC cases (ie, 143 prevalent and 22 incident cases). And the number of EOCRC cases was extended to 347 (ie, 262 prevalent and 85 incident cases) after setting the age cut‐off as 55 years in sensitivity analysis. Furthermore, a total of 719 early‐onset adenomas cases (ie, 472 prevalent and 247 incident cases) were documented in the LRAU‐related analysis.

### 2.5 Genetic susceptibility

To expand the catalog of CRC risk loci, Huyghe et al^15^ and Law et al^16^ have conducted latest GWAS meta‐analyses respectively based on 125 478 and 106 006 individuals of European ancestry and identified 127 CRC risk‐related SNPs (*P* < 5 × 10^−8^, Table S2). All risk variants were free of linkage disequilibrium (LD, *r* ^2^ > .001) and were available in the UK Biobank. We constructed PRS based on the 127 SNPs by summing the product of the number of CRC risk alleles (0, 1 and 2) and corresponding effect size on CRC genetic liability for each risk variant. The CRC PRS was dichotomized as low (≤ the median PRS of non‐CRC cases) and high (above the median PRS of the non‐CRC cases) to assess the genetic risk for early‐onset CRC and its precursory lesion of adenomas.^17^ Three SNPs, including rs35866622, rs601338 and rs281377, which are different from the above 127 CRC risk variants, have been shown to be genetic polymorphisms of *FUT2* gene that regulates gut microbiome.^27^ We obtained the genotyping data of these SNPs in the UK Biobank to test whether the association between LRAU and the risk of EOCRC and its precursory lesion of adenomas is modified by the genetic polymorphisms of *FUT2* gene.

### 2.6 Statistical analysis

The distribution of baseline characteristics of the study population according to colorectal neoplasm status was evaluated in descriptive analyses and differences were tested for statistical significance using the Pearson *χ* ^2^ test for categorical variables (eg, sex) and the *t*‐test for continuous variables (eg, age at recruitment). We used the logistic regression to estimate adjusted odds ratios (ORs) and 95% confidence intervals (95% CIs) for the association of LRAU and other early life factors (Tables S3 and S4) with EOCRC risk. Two levels of statistical adjustments were applied: *model 1* was adjusted for age at recruitment, sex, PRS and the first five principal components, and *model 2* was additionally adjusted for education level, family history of CRC and childhood diabetes. We also investigated whether and to what extent the association between LRAU and EOCRC risk differs by PRS levels (low/high), sex (male/female), family history of CRC (yes/no) and tumor subsites (proximal colon, distal colon, rectum) (Table S5). Here, we used a likelihood ratio test to calculate *P* values for interactions between early‐life factors and the genetic factors.

Moreover, the joint association of LRAU and PRS levels with EOCRC risk was evaluated (Table S6). Furthermore, we conducted gene‐antibiotic interaction analysis to test whether the association between LRAU and EOCRC risk varies by the expression of *FUT2* gene. For the genetic polymorphisms of *FUT2* that were marginally associated with EOCRC risk (ie, rs281377), subgroup analyses were additionally performed based on the rs281377 genotype to assess potential effect modification of the association between LRAU and EOCRC risk by this factor. Also, we estimated the cumulative incidence of EOCRC according to LRAU status by the genetic polymorphisms of *FUT2* gene. Additionally, we used the above analysis to further explore the relationships among LRAU, genetic factors and risk of early‐onset CRC precursors.

We conducted sensitivity analysis to test the robustness of the associations by (a) using multivariable Cox proportional hazards regression to validate the associations regarding LRAU (detail in Supplementary Methods in Data S1, Tables S7 and S8); (b) using 55 years as the age cutoff to define EOCRC (Tables S9 and S10). All analyses were performed in the R software (version 4.1.1), and all statistical tests were two‐sided. Bonferroni correction was applied to account for the multiple testing, where *P*‐value <.005 was regarded as statistically significant and *P*‐value <.05 was regarded as nominally significant.

## 3 RESULTS

### 3.1 Characteristics of the study participants

A total of 113 256 participants were included in the analysis, including 165 EOCRC and 719 EOCRA cases. The distribution of baseline characteristics according to the status of early‐onset CRC and adenomas are summarized in Table 1. Overall, early‐onset CRC and adenomas cases were more likely to be men and have a family history of CRC and a history of LRAU.

**TABLE 1:** Baseline characteristics of study participants.

Characteristic	Non‐EOCRC	EOCRC cases		EOCRA cases	
	(n = 112 209)	(n = 165)	*P*‐value*	(n = 719)	*P*‐value*
Age at recruitment, mean (SD)	55.1 ± 7.7	54.4 ± 7.6	8.75E−09	47.9 ± 5.5	7.50E−06
Gender					
Male	47 033 (41.9)	70 (42.4)	9.57E−01	746 (47.8)	1.51E−03
Female	65 176 (58.1)	95 (57.6)		773 (52.2)	
Education					
College/university degree	51 607 (46.0)	71 (43.0)	7.20E−01	343 (47.7)	1.47E−02
No college/university degree	60 107 (53.6)	93 (56.4)		368 (51.2)	
Missing	3051 (0.4)	1 (0.6)		8 (1.1)	
Family history of CRC					
No	82 801 (73.8)	113 (68.5)	5.74E−08	499 (69.4)	2.20E−16
Yes	7005 (6.2)	28 (17.0)		113 (15.7)	
Missing	22 403 (20.0)	24 (14.5)		107 (14.9)	
Diagnosed with T1DM					
No	111 644 (99.5)	163 (98.8)	5.42E−01	710 (98.7)	1.08E−02
Yes	565 (0.5)	2 (1.2)		9 (1.3)	
PRS^a^					
Low	56 291 (50.2)	53 (32.1)	5.25E−06	264 (36.7)	8.56E−13
High	55 918 (49.8)	112 (67.9)		455 (63.3)	
Continuous	1.1 ± 0.6	1.5 ± 0.6		1.4 ± 0.6	
Long‐term/recurrent antibiotic use					
No	95 658 (85.2)	131 (79.4)	4.45E−02	557 (77.5)	6.46E−09
Yes	16 551 (14.8)	34 (20.6)		162 (22.5)	

^a^ The PRS was categorized as low (≤1.1472) and high (>1.1472) based on the median of PRS in non‐cases.

* *P*‐value from *χ* ^2^ test for categorical variables and from *t*‐test for continuous variables.

### 3.2 Early‐life factors and EOCRC risk

Table 2 shows the results of minimally and fully adjusted ORs for the associations of early‐life factors and PRS with EOCRC risk (for prevalent and incident cases combined and incident cases only). In the fully adjusted model, LRAU was nominally associated with increased EOCRC risk when prevalent and incident cases were analyzed together (OR = 1.48, 95% CI = 1.01‐2.17, *P* = .046). This association was not statistically significant for incident cases only (OR = 1.84, 95% CI = 0.74‐4.61, *P* = .190). Regarding PRS, participants with high genetic risk (vs low) had substantially increased risk of EOCRC (OR = 2.00, 95% CI = 1.68‐2.38, *P* < .001 for incident and prevalent cases combined and OR = 2.49, 95% CI = 1.61‐3.85, *P* < .001 for incident cases only), which remained after Bonferroni correction for multiple testing. When PRS was grouped into tertiles, participants in the middle (OR = 1.29, 95% CI = 1.02‐1.63, *P* = .032) and upper tertile (OR = 2.41, 95% CI = 1.95‐2.97, *P* < .001) had significantly increased EOCRC risk when compared to those in the lowest tertile (*P*_Trend_ < .001, data not shown).

**TABLE 2:** Associations of LRAU during early life and polygenic risk score with risk of early‐onset colorectal neoplasm.

	Cases	Incident + prevalent cases				Cases	Incident cases			
		Model 1		Model 2			Model 1		Model 2	
		OR (95% CI)	*P*	OR (95% CI)	*P*		OR (95% CI)	*P*	OR (95% CI)	*P*
*Early‐onset colorectal cancer*										
LRAU during early life										
No	131	1.00 (Ref)		1.00 (Ref)		15	1.00 (Ref)		1.00 (Ref)	
Yes	34	1.48 (1.01‐2.17)	.044	1.48 (1.01‐2.17)	.046	7	1.88 (0.76‐4.66)	.175	1.84 (0.74‐4.61)	.190
Polygenetic risk score (PRS)										
Low genetic risk	200	1.00 (Ref)		1.00 (Ref)		29	1.00 (Ref)		1.00 (Ref)	
High genetic risk	394	2.03 (1.71‐2.41)	<.001	2.00 (1.68‐2.38)	<.001	72	2.51 (1.62‐3.88)	<.001	2.49 (1.61‐3.85)	<.001
Continuous	594	2.33 (2.02‐2.69)	<.001	2.29 (1.98‐2.64)	<.001	101	2.54 (1.79‐3.60)	<.001	2.51 (1.77‐3.56)	<.001
*Early‐onset colorectal adenomas*										
LRAU during early life										
No	557	1.00 (Ref)		1.00 (Ref)		195	1.00 (Ref)		1.00 (Ref)	
Yes	162	1.41 (1.18‐1.68)	<.001	1.40 (1.17‐1.68)	<.001	52	1.16 (0.85‐1.58)	.351	1.15 (0.84‐1.57)	.382
Polygenetic risk score (PRS)										
Low genetic risk	920	1.00 (Ref)		1.00 (Ref)		336	1.00 (Ref)		1.00 (Ref)	
High genetic risk	1430	1.52 (1.40‐1.65)	<.001	1.50 (1.38‐1.63)	<.001	495	1.43 (1.25‐1.65)	<.001	1.41 (1.23‐1.63)	<.001
Continuous	2350	1.62 (1.51‐1.75)	<.001	1.59 (1.48‐1.71)	<.001	831	1.59 (1.40‐1.80)	<.001	1.57 (1.39‐1.77)	<.001

*Note*: Model 1: adjusted for age, sex, PRS and the top five principal components. Models for genetic risk were not adjusted for PRS. Model 2: adjusted for age, sex, education(college/non‐college), family history of CRC (yes/no), type 1 diabetes(yes/no), PRS and the top five principal components. Models for genetic risk were not adjusted for PRS.

Subgroup analyses of the association between LRAU and EOCRC risk according to PRS (high/low, Table 3) showed positive association at a nominal level between LRAU and EOCRC risk among participants with high PRS (OR = 1.72, 95% CI = 1.09‐2.71, *P* = .019), whereas no association was observed among those with low PRS (OR = 1.05, 95% CI = 0.51‐2.18, *P* = .889), whilst the result of interaction test was not statistically significant (*P*_Interaction_ = .434). Table S6 shows results of the joint association of LRAU and PRS with EOCRC risk. Compared to participants with low PRS and no LRAU, the risk of EOCRC was highest among those with high PRS and LRAU (OR = 3.17, 95% CI = 1.93‐5.21, *P* < .001), followed by those with high PRS and no LRAU (OR = 1.98, 95% CI = 1.38‐2.84, *P* < .001). No significant associations were observed between the other early‐life factors and EOCRC risk overall and according to polygenic risk (Tables S3 and S4).

**TABLE 3:** Association between LRAU during early life and risk of early‐onset colorectal neoplasm by polygenic risk score.

	Cases	Low genetic risk				Cases	High genetic risk				*P*_interaction_
		Model 1		Model 2			Model 1		Model 2		
		OR (95% CI)	*P*	OR (95% CI)	*P*		OR (95% CI)	*P*	OR (95% CI)	*P*	
*EOCRC*											
LRAU during early life											
No	131	1.00 (Ref)		1.00 (Ref)		15	1.00 (Ref)		1.00 (Ref)		
Yes	34	1.06 (0.51‐2.19)	.872	1.05 (0.51‐2.18)	.889	7	1.71 (1.09‐2.70)	.020	1.72 (1.09‐2.71)	.019	.434
*EOCRA*											
LRAU during early life											
No	204	1.00 (Ref)		1.00 (Ref)		353	1.00 (Ref)		1.00 (Ref)		
Yes	60	1.43 (1.06‐1.91)	.018	1.41 (1.05‐1.90)	.021	102	1.39 (1.11‐1.74)	.004	1.39 (1.11‐1.74)	.005	.993

*Note*: Model 1: adjusted for age, sex and the top five principal components. Model 2: adjusted for age, sex, education(college/non‐college), family history of CRC (yes/no), type 1 diabetes(yes/no) and the top five principal components.

Further stratification of the association between LRAU and EOCRC risk by sex, family history of CRC and tumor subsite (Table S5) showed nominally significant associations with LRAU in women (OR = 1.60, 95% CI = 1.00‐2.53, *P* = .047) and those with family history of CRC (OR = 2.34, 95% CI = 1.01‐5.43, *P* = .047). In sensitivity analysis using 55 years as the age cutoff to define EOCRC (Tables S9 and S10), the association between LRAU and EOCRC risk remained stable among participants with high genetic risk (OR = 1.49, 95% CI = 1.08‐2.05, *P* = .015; Table S10).

For the precursor lesion of CRC, LRAU was significantly associated with increased risk of EOCRA (OR = 1.40, 95% CI = 1.17‐1.68, *P* < .001 for incident and prevalent cases combined, Table 2), regardless of genetic liability (OR = 1.41, 95% CI = 1.05‐1.90, *P* = .021 for low and OR = 1.39, 95% CI = 1.11‐1.74, *P* = .005 for high PRS group, Table 3). Again, the association between LRAU and EOCRA risk was not statistically significant for incident cases only (OR = 1.15, 95% CI = 0.84‐1.57, *P* = .402). Sensitivity analysis using multivariable Cox proportional hazards regression showed very similar associations between LRAU and risk of EOCRC and EOCRA overall and by genetic risk (Tables S7 and S8). In sensitivity analysis where the initial follow‐up age of EOCRC was set to 19 years in the LRAU‐related analysis, similar results as those reported for the main analysis were observed (Supplementary Methods in Data S1, data not shown).

### 3.3 Gene‐antibiotic interactions for EOCRC risk

In analysis of the three genetic polymorphisms of *FUT2* gene (ie, rs35866622, rs601338 and rs281337) in their associations with EOCRC risk, we observed no associations of rs35866622 and rs601338 with EOCRC risk, but a marginal association for rs281377 (OR = 1.23, 95% CI = 0.99‐1.54, *P* = .068) (Table S11). Risk estimates appeared much greater for LRAU with EOCRC risk in participants with rs281377 TT genotype (OR = 2.73, 95% CI = 0.83‐8.95, *P* = .098) than those with CT and TT genotype (Table 4), although none of the estimates reached statistical significance. However, there were also strong positive associations between LRAU and the EOCRA in the rs281377 TT (OR = 1.75, 95% CI: 1.16‐2.64, *P* = .008) and CT genotypes (OR = 1.51, 95% CI: 1.17‐1.94, *P* = .001), whereas no significant association was observed among individuals with rs281377 CC genotype (OR = 1.10, 95% CI = 0.79‐1.52, *P* = .587). Table 4 shows the adjusted ORs for the association between LRAU and risk of EOCRC as well as EOCRA, respectively, stratified by the rs281377 genotypes of *FUT2*.

**TABLE 4:** Effect of antibiotic use during early life on risk of early‐onset neoplasm stratified by the genotype in the UK Biobank.

FUT2 genotype status	Cases/noncases	Model 1		Model 2		Log‐rank *P*
		OR (95% CI)	*P* value	OR (95% CI)	*P* value	
EOCRC (cases, n = 165)						
rs281377 CC						.100
No LRAU during early life	42/31 050	1.00 (Ref)		1.00 (Ref)		
LRAU during early life	12/5274	1.61 (0.84‐3.09)	.153	1.59 (0.83‐3.05)	.164	
rs281377 CT						.300
No LRAU during early life	79/46 896	1.00 (Ref)		1.00 (Ref)		
LRAU during early life	18/8157	1.28 (0.76‐2.16)	.348	1.27 (0.75‐2.14)	.370	
rs281377 TT						.200
No LRAU during early life	10/17 712	1.00 (Ref)		1.00 (Ref)		
LRAU during early life	4/3120	2.73 (0.83‐8.95)	.098	2.74 (0.84‐9.01)	.096	
EOCRA (cases, n = 719)						
rs281377 CC						.070
No LRAU during early life	197/31 050	1.00 (Ref)		1.00 (Ref)		
LRAU during early life	45/5274	1.10 (0.79‐1.52)	.586	1.10 (0.79‐1.52)	.587	
rs281377 CT						<.001
No LRAU during early life	271/46 896	1.00 (Ref)		1.00 (Ref)		
LRAU during early life	85/8157	1.52 (1.19‐1.95)	.001	1.51 (1.17‐1.94)	.001	
rs281377 TT						<.001
No LRAU during early life	89/17 712	1.00 (Ref)		1.00 (Ref)		
LRAU during early life	32/3120	1.74 (1.15‐2.63)	.008	1.75 (1.16‐2.64)	.008	

*Note*: Model 1: adjusted for age, sex and the top five principal components. Model 2: adjusted for age, sex, education(college/non‐college), family history of CRC (yes/no), type 1 diabetes(yes/no) and the top five principal components.

## 4 DISCUSSION

Given that CRC takes several decades to develop, investigating early‐life factors as potential risk factors for EOCRC is important. We investigated the association between LRAU during early life as well as its interactions and joint associations with genetic risk factors. Results suggested that LRAU was associated with increased risk of both EOCRC and EOCRA. In participants with high PRS and family history of CRC, LRAU was associated with 72% and 134% higher risk of EOCRC, respectively. In addition, when stratified by genetic polymorphisms of *FUT2*, LRAU appeared to confer a comparatively greater risk for EOCRA among participants with rs281377 TT/CT genotype.

The observed higher EOCRC risk among those with a history of LRAU during childhood lends credence to the findings of a case‐control study that showed that antibiotic exposure was strongly associated with EOCRC risk, whereas a modest association was observed with LOCRC risk.^11^ However, the authors did not assess the effect of early‐life LRAU and whether the association was modified by genetic factors.^11^ Our study suggested that the risk of EOCRC with LRAU appeared to be restricted to those with high PRS and family history of CRC but we observed no significant interaction between LRAU and genetic factors. Studying EOCRA helped us understand etiologic contributors to the rising EOCRC incidence. A previous study demonstrated a positive association between long‐term use of antibiotics and risk of adenomas.^9^ Our analysis showed consistent results and further supports the negative role of LRAU during early life in the etiology and development of EOCRC. Given majority of colorectal adenomas are asymptomatic and only detected by screening, the associations between exposures and adenomas can be confounded by colonoscopy.^28^ However, antibiotics use appeared to be independent of colonoscopy or related symptoms. Notably, LRAU appeared to increase risk of CRC precursors regardless of genetic liability, whereas the increased EOCRC risk was restricted to high PRS group. The latter probably suggested that inadequate power due to a few cases might lead to difference in LRAU with EOCRC risk by PRS levels.

Genetic factors play an important role in risk of early‐onset colorectal neoplasm, and it has been shown that the association between genetic factor (eg, PRS) and CRC risk is particularly strong for EOCRC.^17^ However, genetic factors are not modifiable and there is limited evidence supporting differential CRC screening among younger adults with high genetic risk such as those with a family history of CRC.^29^ Hence, identification of modifiable factors that independently interact with genetic factors to affect EOCRC risk is highly relevant. Our findings suggested that individuals with genetic risk factors (ie, family history of CRC) who have experienced early‐life antibiotics use on a long‐term basis are probably at increased EOCRC risk. Given that antibiotics remain valuable in the management of bacterial infections during early life, investigating the pros and cons of early‐life antibiotic use is of great significance. Consistent with our findings, animal studies have supported a potential detrimental role for antibiotics in microbiome‐related pathways of CRC.^30^, ^31^ However, another study has reported that metronidazole (a type of antibiotic) reduced *Fusobacterium* load, cancer cell proliferation and overall tumor growth of mouse with xenograft.^32^ Therefore, influence of antibiotics on colorectal carcinogenesis is probably bidirectional and multiple potential mechanisms remain to be explored. Our findings, however, indicated that novel strategies are still expected to substitute or complement antibiotic therapies, so that pathogens can be targeted selectively without perturbing the microbiota and the beneficial effects they confer to the host.^33^ Notably, pathogens necessitating antibiotics use may induce inflammation, a well‐established risk factor for CRC, and may mediate the observed association between antibiotics use and EOCRC risk.^34^

Besides, a meta‐analysis of epidemiological studies involving participants of all ages, for whom the majority are usually older than 60 years, have demonstrated that the association between LRAU and CRC risk is restricted to broad‐spectrum antibiotics.^35^ We could not investigate whether this also holds for EOCRC risk, as data on type of antibiotics used during childhood/adolescence were not available in the UK Biobank. Further studies investigating whether the association between LRAU and EOCRC risk varies by the type of antibiotics are thus warranted and findings from such studies may be valuable for recommendations for early‐life antibiotic use in individuals with high genetic risk. In addition, the use of antibiotics early in life, particularly during childhood, could be susceptible to recall bias and may leading to a misclassification of this exposure. The reported antibiotics usage in present study likely reflected consumption during adolescence, a period when participants' recollections were likely to be more reliable, rather than during childhood. Therefore, a precise medical record of antibiotic usages detailing exact timing and early‐life stage of administration is essential for further validation. In general, our study extended the findings of prior studies by demonstrating how LRAU during early life as well as genetic factors modify the risk of early‐onset CRC and precursors, providing new thoughts for understanding the etiology of EOCRC regarding gut microbiota regulation. Even though these, to our knowledge, have not been investigated previously, the mechanism and clinical relevance of these findings warrant further study and validation.

This observed associations of LRAU with risk of EOCRC and EOCRA as well as their variation by genetic risk factors are biologically plausible and may be largely explained by the impact on gut microbiota. For example, LRAU may cause long‐standing changes in the gut microbiota and impede normal immune surveillance caused by irregularities in immunostimulatory bacterial products (eg, adhesins expressed by *Fusobacterium nucleatum* engage an immunoreceptor on immune cells to block their cytotoxic activity on tumor cells and dysregulating antitumor immunity^36^, ^37^), thereby increasing CRC risk.^19^ Also, alterations in the normal gut commensals may allow colonization of pathogenic bacteria (eg, *Escherichia coli*, *Bacteroides fragilis* and *Fusobacterium nucleatum*), which can invade and damage the gut mucosa and lead to inflammation and tumor initiation.^38^, ^39^, ^40^, ^41^, ^42^ *FUT2* is one of the enzymes that are responsible for the addition of fucose to proteins or lipids by α‐1,2‐fucosylation on the intestinal mucosa, which can serve as attachment site and carbon source for intestinal bacteria.^20^ The *FUT2* gene encodes the *galactoside 2‐α‐l‐fucosyltransferase 2* enzyme and thus influences several types of host‐microbial interactions.^21^ In‐vivo studies have also shown that gut microbiota in *FUT2*‐deficient mice is altered structurally and functionally (eg, increased proportional representation of several genera of *Bacteroidetes* and decreased alpha diversity).^21^, ^43^, ^44^ This increases the generation of lysophosphatidylcholine, which in turn promotes inflammation and epithelial barrier damage.^45^

Our study has several strengths. To our knowledge, it is the first to investigate the effect of LRAU during early life in EOCRC risk. The early‐onset precursor lesion of CRC was also studied to further support the role of LRAU in the etiology of EOCRC. We were particularly able to evaluate potential effect modification and joint associations by genetic risk factors, including family history of CRC, PRS and the rs281377 genotype. Moreover, cases of early‐onset colorectal neoplasm in our study were ascertained via linkage to national registries and inpatient medical records, minimizing misclassification. However, some limitations need to be acknowledged. First, information of early‐life LRAU is accessible only for a specific subgroup within the full cohort. It is thus possible that the analytic sample differs from the general population, as is also the case with the UK Biobank.^46^ Also, we included participants of European descent; hence, the findings may not be generalizable to populations of other ancestries. Second, given that the participants had to recall events from their childhood and adolescence, information regarding very early life exposures (ie, antibiotics use for tonsillitis during childhood) could be subject to recall bias and may leading to a misclassification, especially for prevalent cases which have been diagnosed before the data collection in baseline. The reported antibiotics usage in our study likely reflected consumption during adolescence rather than childhood, owing to the reliability of participants' recollections. Besides, LRAU during early life broadly covered both long‐term use and recurrent courses of antibiotics while more detailed description was not available, and it is difficult to estimate the exact time or early‐life period of antibiotic use. Third, even though we adjusted for several potential confounding factors (eg, sex, education level, family history of CRC and PRS), residual confounding remains possible due to unmeasured and/or imperfectly ascertained factors. For example, we lacked information on lifestyle factors during early life such as diet, alcohol consumption and body mass index, which have been reported to be associated with EOCRC risk.^12^, ^13^, ^14^ Fourth, it would be of interest to also assess dose‐response relationship between LRAU and EOCRC risk and whether the association between LRAU and EOCRC risk varies by type of antibiotics, the duration of antibiotics use as well as the reason of antibiotic use, but we lacked information on these important variables. Finally, the cases for gene‐antibiotic interaction analysis were somewhat limited. To solve this, we included prevalent cases to increase the statistical power. Since all the cases occurred after age 30, it could be inferred that the exposures of interest preceded the outcome and similar patterns of associations were observed when we analyzed the incident cases separately. However, it should be noted that this might induce Neyman bias if the exposure was associated with survival other than EOCRC risk.

## 5 CONCLUSIONS

Our study indicated that LRAU during early life was associated with an increased risk of early‐onset CRC (at a nominal level) and adenomas, and the association for adenomas was predominantly among individuals with rs281377 TT/CT genotype. This suggests that LRAU during early life as well as genetic factors may modify the risk of EOCRC and adenomas, offering new prospective for understanding the etiology of EOCRC regarding gut microbiota regulation. Given the potential for recall bias and misclassification, additional research detailing the type, duration and specific early‐life period of antibiotics use is required to provide valuable insights for early life clinical practice. Moreover, further studies are warranted to explore how early‐life antibiotic use and genetic factors contribute together to modify EOCRC risk, particularly concerning the microbiome‐related pathway underlying colorectal carcinogenesis.

## AUTHOR CONTRIBUTIONS

**Fangyuan Jiang:** Performed the literature review and the data analyses; Wrote the first draft; Article was revised and edited. **Daniel Boakye:** Wrote the first draft. **Shuai Yuan:** article was revised and edited. **Mingyang Song:** Article was revised and edited. **Susanna C. Larsson:** Article was revised and edited. **Edward L Giovannucci:** article was revised and edited. **Evropi Theodoratou:** Conceptualized the project. **Kefeng Ding:** Conceptualized the project. **Xue Li:** Conceptualized the project; article was revised and edited; study guarantor. All authors critically reviewed the article for important intellectual content. The corresponding author attests that all listed authors meet authorship criteria and that no others meeting the criteria have been omitted. The work reported in the article has been performed by the authors, unless clearly specified in the text.

## FUNDING INFORMATION

XL: the Natural Science Fund for Distinguished Young Scholars of Zhejiang Province (LR22H260001) and the National Nature Science Foundation of China (No. 82204019); ET: CRUK Career Development Fellowship (C31250/A22804); KFD: Project of the regional diagnosis and treatment center of the Health Planning Committee (No. JBZX‐201903); SCL: the Swedish Heart‐Lung Foundation (Hjärt‐Lungfonden, 20210351), the Swedish Research Council (Vetenskapsrådet, 2019‐00977) and the Swedish Cancer Society (Cancerfonden); YSZ: the National Natural Science Foundation of China (No. 82103905); YZH: Natural Science Foundation of Sichuan (82103918) and Sichuan Provincial Nature Science Foundation (2022NSFSC1314).

## CONFLICT OF INTEREST STATEMENT

Daniel Boakye is now employed by PMI Global Studio Limited. All the other authors declare no competing interest. “All authors have completed the ICMJE uniform disclosure form at http://www.icmje.org/disclosure-of-interest/ and declare: no support from any organization for the submitted work; no financial relationships with any organizations that might have an interest in the submitted work in the previous 3 years; no other relationships or activities that could appear to have influenced the submitted work.”

## ETHICS STATEMENT

Ethical approval was granted by the UK Biobank by the North West‐Haydock Research Ethics Committee (REC reference: 16/NW/0274). Informed consent was obtained from all participants prior to enrollment and the study was performed in accordance with the Declaration of Helsinki.

## Supporting information


**Figure S1.** Flowchart of study population selection.

**Table S1.** Early‐life factors were assessed in the UKB baseline questionnaire.
**Table S2.** Colorectal cancer variants used for the generation of PRS.
**Table S3.** Associations of early‐life factors and polygenic risk score with EOCRC risk.
**Table S4.** Association between early‐life factors and EOCRC risk by polygenic risk score.
**Table S5.** Association between early‐life factors and risk of early‐onset colorectal neoplasm by gender, family history and anatomic sites.
**Table S6.** Risk of early‐onset neoplasm by joint categorization for genetic risk and LRAU during early life.
**Table S7.** Associations of LRAU during early life and polygenic risk score with early‐onset colorectal neoplasm risk using Cox regression.
**Table S8.** Association between LRAU during early life and risk of early‐onset colorectal neoplasm by polygenic risk score using Cox regression.
**Table S9.** Associations of LRAU and polygenic risk score with EOCRC risk after using age 55 as the cutoff to define EOCRC.
**Table S10.** Association between LRAU and EOCRC risk by polygenic risk score after using age 55 as the cutoff to define EOCRC.
**Table S11.** Gene‐antibiotic interaction estimates for the risk of early‐onset colorectal neoplasm.

## Data Availability

This research was conducted using the UK Biobank study under Application Number 66354. The UK Biobank is an open access resource and bona fide researchers can apply to use the UK Biobank dataset by registering and applying at http://ukbiobank.ac.uk/register-apply/. Further information is available from the corresponding author upon request.
