# Supplementary material for: Association between antibiotic use during early life and early‐onset colorectal cancer risk overall and according to polygenic risk and FUT2 genotypes
Source: Int J Cancer. 2023 Jul 28;153(9):1602–11. doi: 10.1002/ijc.34648 (PMC10953323; doi:10.1002/ijc.34648)
Supplement: Supplementary file 1 — Figure S1. Flowchart of study population selection. [file IJC-153-1602-s002.pdf]

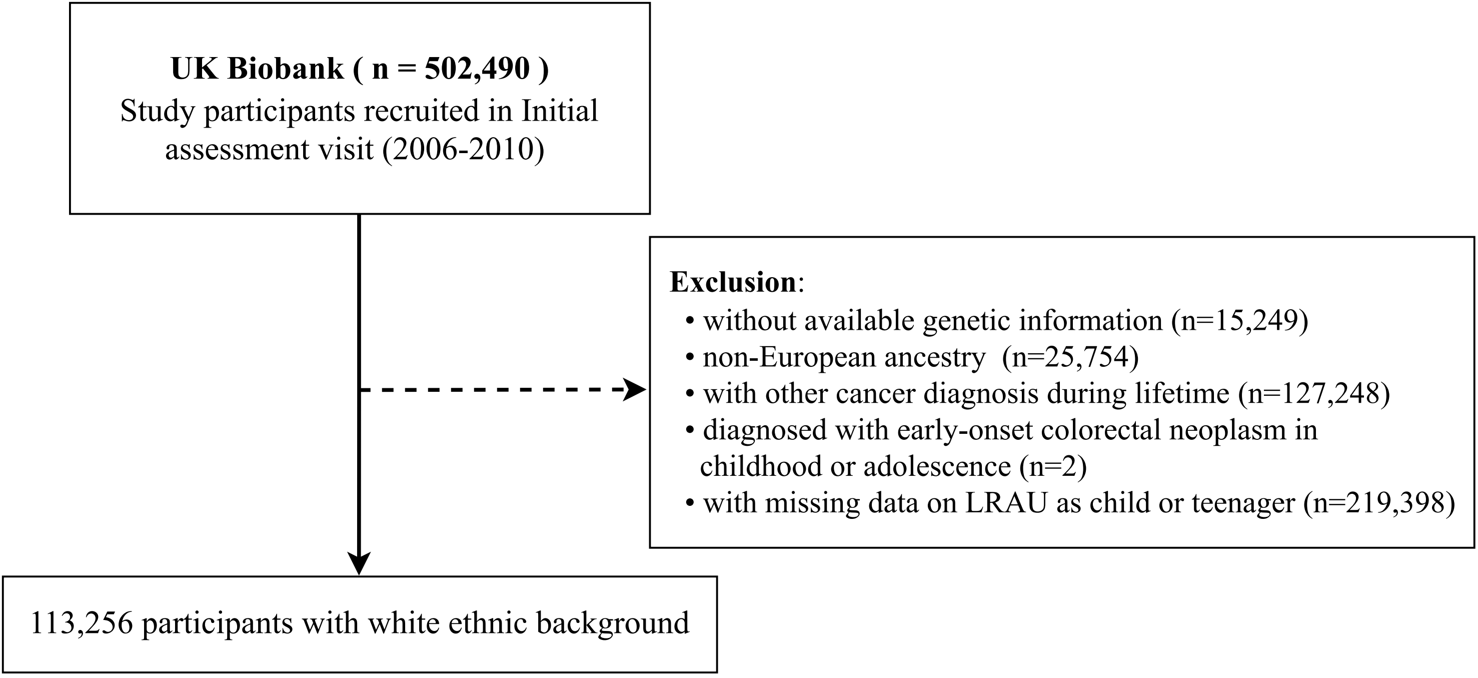
UK Biobank (n = 502,490)
Study participants recruited in Initial assessment visit (2006-2010)
Exclusion:
• without available genetic information (n=15,249)
• non-European ancestry (n=25,754)
• with other cancer diagnosis during lifetime (n=127,248)
• diagnosed with early-onset colorectal neoplasm in childhood or adolescence (n=2)
• with missing data on LRAU as child or teenager (n=219,398)
113,256 participants with white ethnic background
