# Supplementary material for: Association between antibiotic use during early life and early‐onset colorectal cancer risk overall and according to polygenic risk and FUT2 genotypes
Source: Int J Cancer. 2023 Jul 28;153(9):1602–11. doi: 10.1002/ijc.34648 (PMC10953323; doi:10.1002/ijc.34648)
Supplement: Supplementary file 2 — Table S1. Early‐life factors were assessed in the UKB baseline questionnaire. Table S2. Colorectal cancer variants used for the generation of PRS. Table S3. Associations of early‐life factors and polygenic risk score with EOCRC risk. Table S4. Association between early‐life factors and EOCRC risk by polygenic risk score. Table S5. Association between early‐life factors and risk of early‐onset colorectal neoplasm by gender, family history and anatomic sites. Table S6. Risk of early‐onset neoplasm by joint categorization for genetic risk and LRAU during early life. Table S7. Associations of LRAU during early life and polygenic risk score with early‐onset colorectal neoplasm risk using Cox regression. Table S8. Association between LRAU during early life and risk of early‐onset colorectal neoplasm by polygenic risk score using Cox regression. Table S9. Associations of LRAU and polygenic risk score with EOCRC risk after using age 55 as the cutoff to define EOCRC. Table S10. Association between LRAU and EOCRC risk by polygenic risk score after using age 55 as the cutoff to define EOCRC. Table S11. Gene‐antibiotic interaction estimates for the risk of early‐onset colorectal neoplasm. [file IJC-153-1602-s001.docx]

**Association between antibiotic use during early life and early-onset colorectal cancer risk overall and according to polygenic risk and FUT2 genotypes**

Fangyuan Jiang*, Daniel Boakye*, Jing Sun, Lijuan Wang, Lili Yu, Xuan Zhou, Jianhui Zhao, Zilong Bian, Peige Song, Yazhou He, Yingshuang Zhu, Jie Chen, Shuai Yuan, Mingyang Song, Susanna C. Larsson, Edward L Giovannucci, Evropi Theodoratou^#^, Kefeng Ding^#^, Xue Li^#^

**Summary of contents**

Supplementary Methods

Supplementary Table 1: Early-life factors assessed in the UKB baseline questionnaire.

Supplementary Table 2: Colorectal cancer variants used for the generation of PRS.

Supplementary Table 3: Associations of early-life factors and polygenic risk score with EOCRC risk.

Supplementary Table 4: Association between early-life factors and EOCRC risk by polygenic risk score.

Supplementary Table 5: Association between early-life factors and risk of early-onset colorectal neoplasm by gender, family history, and anatomic sites.

Supplementary Table 6: Risk of Early-Onset Neoplasm by joint categorization for genetic risk and LRAU during early life.

Supplementary Table 7: Associations of LRAU during early life and polygenic risk score with early-onset colorectal neoplasm risk using cox regression.

Supplementary Table 8: Association between LRAU during early life and risk of early-onset colorectal neoplasm by polygenic risk score using cox regression.

Supplementary Table 9: Associations of LRAU and polygenic risk score with EOCRC risk after using age 55 as the cutoff to define EOCRC.

Supplementary Table 10: Association between LRAU and EOCRC risk by polygenic risk score after using age 55 as the cutoff to define EOCRC.

Supplementary Table 11: Gene-antibiotic interaction estimates for the risk of Early-onset Colorectal Neoplasm.

Supplementary Figure 1. Flowchart of study population selection.

**Supplementary Methods**

Minimally adjusted multivariable Cox models included age at recruitment, sex, PRS, and the first 5 principal components. Fully adjusted models also included education level, family history of CRC, and childhood diabetes. Follow-up time (in years) was calculated from birth until date of first EOCRC diagnosis (incidence/prevalence), death, loss to follow-up, or end of follow-up (50th birthday), whichever occurred first. We also conducted sensitivity analysis setting the initial follow-up age as 19 years in the LRAU-related analysis to validate the association (data not shown). Schoenfeld residuals method showed that the proportional hazards assumption was not violated.

**Supplementary Table 1: Early-life factors assessed in the UKB baseline questionnaire.**

| **Early-life factors** | | **Field ID** | **Notes** | **Sources** |
| --- | --- | --- | --- | --- |
| long-term antibiotics use during early life | | 21067 | "During childhood or as a teenager did you receive long-term or recurrent courses (3 or more per year) of antibiotics (for example for tonsillitis or acne)?" | Online follow-up |
| Birth weight | | 20022 | Participants were asked to enter their own birth weight. | Verbal interview |
| Part of a multiple birth | | 1777 | "Are you a twin, triplet or other multiple birth?" | Touchscreen |
| Breastfeeding in infancy | | 1677 | "Were you breastfed when you were a baby?" | Touchscreen |
| Comparative body size to peers at age 10 years | | 1687 | "When you were 10 years old, compared to average would you describe yourself as about average, thinner or plumper?” | Touchscreen |
| Childhood sunburn occasions | | 1737 | "Before the age of 15, how many times did you suffer sunburn that was painful for at least 2 days or caused blistering?" | Touchscreen |
| Female only | Age at menarche | 2714 | "How old were you when your periods started?" | Touchscreen |
|  | Menstrual cycle | 3710 | "How many days is your usual menstrual cycle? (The number of days between each menstrual period)" | Touchscreen |
| Male only | Relative age of first facial hair | 2375 | "When did you start to grow facial hair?" | Touchscreen |
|  | Relative age voice broke | 2385 | "When did your voice break?" | Touchscreen |

**Supplementary Table 2: Colorectal cancer variants used for the generation of PRS.**

| SNP | Chr | Position | Effect allele  /Other allele | EAF | Weight （beta） | *P* value |
| --- | --- | --- | --- | --- | --- | --- |
| rs10049390 | 3 | 133701119 | A/G | 0.71 | 0.058 | 3.79E-09 |
| rs1078643 | 17 | 10707241 | A/G | 0.75 | 0.077 | 6.63E-12 |
| rs10821907 | 10 | 52648454 | C/T | 0.83 | 0.077 | 5.00E-10 |
| rs10980628 | 9 | 113671403 | C/T | 0.21 | 0.068 | 2.77E-09 |
| rs11087784 | 20 | 7740976 | G/A | 0.15 | 0.086 | 2.70E-13 |
| rs11196170 | 10 | 114722621 | A/G | 0.22 | 0.058 | 3.30E-07 |
| rs11610543 | 12 | 43134191 | G/A | 0.52 | 0.049 | 1.30E-09 |
| rs11874392 | 18 | 46453156 | A/T | 0.55 | 0.157 | 3.80E-74 |
| rs11884596 | 2 | 199612407 | C/T | 0.39 | 0.058 | 3.62E-09 |
| rs12144319 | 1 | 55246035 | C/T | 0.29 | 0.068 | 3.27E-11 |
| rs12149163 | 16 | 86339315 | T/C | 0.5 | 0.049 | 5.40E-09 |
| rs12246635 | 10 | 114288619 | C/T | 0.1 | 0.095 | 4.90E-12 |
| rs12372718 | 12 | 51171090 | G/A | 0.39 | 0.086 | 1.90E-23 |
| rs12514517 | 5 | 40280076 | A/G | 0.29 | 0.095 | 3.70E-21 |
| rs12672022 | 7 | 45136423 | T/C | 0.84 | 0.068 | 2.78E-08 |
| rs13149359 | 4 | 94938618 | A/C | 0.37 | 0.049 | 1.20E-08 |
| rs1391441 | 4 | 106128760 | A/G | 0.65 | 0.049 | 1.58E-08 |
| rs143635270 | 16 | 9242047 | G/A | 0.99 | 0.068 | 1.20E-01 |
| rs17011141 | 1 | 222112634 | G/A | 0.21 | 0.086 | 6.10E-16 |
| rs17094983 | 14 | 59189361 | G/A | 0.88 | 0.086 | 4.61E-11 |
| rs174533 | 11 | 61549025 | G/A | 0.67 | 0.068 | 1.20E-11 |
| rs189583 | 20 | 6376457 | G/C | 0.33 | 0.095 | 1.20E-22 |
| rs2186607 | 11 | 101656397 | T/A | 0.5 | 0.049 | 1.47E-09 |
| rs2250430 | 12 | 6421174 | T/A | 0.71 | 0.068 | 3.30E-10 |
| rs2516420 | 6 | 31449620 | C/T | 0.93 | 0.113 | 2.01E-10 |
| rs2735940 | 5 | 1296486 | G/A | 0.5 | 0.086 | 5.10E-25 |
| rs28488 | 20 | 6762221 | T/C | 0.67 | 0.068 | 2.60E-14 |
| rs28840750 | 19 | 33519927 | T/G | 0.95 | 0.191 | 3.70E-23 |
| rs3125049 | 6 | 160754712 | G/A | 0.85 | 0.02 | 5.10E-02 |
| rs3133285 | 8 | 117629411 | G/C | 0.82 | 0.068 | 6.00E-10 |
| rs3217810 | 12 | 4388271 | T/C | 0.13 | 0.122 | 3.60E-19 |
| rs3217874 | 12 | 4400808 | T/C | 0.43 | 0.058 | 2.38E-09 |
| rs34405347 | 9 | 101679752 | T/G | 0.9 | 0.086 | 3.06E-08 |
| rs34797592 | 19 | 16417198 | T/C | 0.11 | 0.086 | 4.15E-10 |
| rs35107139 | 14 | 54419106 | C/A | 0.42 | 0.086 | 1.80E-22 |
| rs35470271 | 3 | 40915239 | G/A | 0.15 | 0.095 | 1.20E-16 |
| rs35808169 | 12 | 4368607 | C/T | 0.17 | 0.077 | 1.50E-11 |
| rs4313119 | 8 | 128571855 | G/T | 0.75 | 0.058 | 2.13E-09 |
| rs448513 | 2 | 159964552 | C/T | 0.35 | 0.049 | 4.44E-08 |
| rs4759277 | 12 | 57533690 | A/C | 0.33 | 0.049 | 9.38E-09 |
| rs4917993 | 10 | 104791595 | C/G | 0.87 | 0.049 | 1.70E-04 |
| rs4968127 | 17 | 809643 | G/A | 0.37 | 0.068 | 1.30E-12 |
| rs4976270 | 5 | 134467220 | C/T | 0.55 | 0.068 | 4.80E-15 |
| rs55990915 | 12 | 117763309 | A/C | 0.13 | 0.068 | 1.80E-06 |
| rs56324967 | 15 | 67402824 | C/T | 0.65 | 0.068 | 1.14E-13 |
| rs58658771 | 15 | 33001734 | A/T | 0.19 | 0.14 | 6.00E-37 |
| rs597808 | 12 | 111973358 | G/A | 0.52 | 0.077 | 2.60E-16 |
| rs6063514 | 20 | 49055318 | C/T | 0.61 | 0.068 | 7.60E-13 |
| rs62396735 | 6 | 41702582 | C/T | 0.29 | 0.03 | 6.30E-04 |
| rs62404966 | 6 | 55712124 | C/T | 0.76 | 0.058 | 2.60E-09 |
| rs6678517 | 1 | 183002639 | A/G | 0.59 | 0.077 | 2.40E-16 |
| rs6781752 | 3 | 66365163 | A/G | 0.21 | 0.058 | 7.10E-08 |
| rs6983267 | 8 | 128413305 | G/T | 0.52 | 0.148 | 3.40E-64 |
| rs704017 | 10 | 80819132 | G/A | 0.58 | 0.077 | 5.20E-18 |
| rs7121958 | 11 | 74280012 | G/T | 0.51 | 0.077 | 1.40E-20 |
| rs7160450 | 14 | 54457231 | T/C | 0.38 | 0.058 | 4.00E-10 |
| rs72942485 | 3 | 112999560 | G/A | 0.98 | 0.174 | 2.08E-08 |
| rs7300312 | 12 | 115890922 | C/T | 0.57 | 0.068 | 7.50E-14 |
| rs73068325 | 19 | 59079096 | T/C | 0.18 | 0.068 | 4.25E-08 |
| rs7333607 | 13 | 37462010 | G/A | 0.22 | 0.077 | 6.32E-13 |
| rs75610640 | 2 | 192562764 | C/T | 0.16 | 0.039 | 1.00E-03 |
| rs75954926 | 17 | 81061048 | G/A | 0.64 | 0.086 | 2.96E-18 |
| rs7708610 | 5 | 40102443 | A/G | 0.35 | 0.058 | 3.78E-09 |
| rs78341008 | 13 | 73791554 | C/T | 0.07 | 0.113 | 3.21E-10 |
| rs9271695 | 6 | 32593080 | G/A | 0.81 | 0.086 | 1.12E-13 |
| rs983318 | 17 | 70413253 | A/G | 0.24 | 0.058 | 5.57E-09 |
| rs983402 | 2 | 199781586 | T/C | 0.3 | 0.068 | 7.71E-12 |
| rs9924886 | 16 | 68743939 | A/C | 0.73 | 0.058 | 3.10E-08 |
| rs994308 | 20 | 6603622 | C/T | 0.58 | 0.058 | 8.57E-12 |
| rs10152518 | 15 | 68177162 | G/A | 0.19 | 0.077 | 3.24E-08 |
| rs10161980 | 13 | 34093518 | C/G | 0.62 | 0.058 | 1.96E-08 |
| rs10849432 | 12 | 6385727 | T/C | 0.90 | 0.058 | 6.89E-04 |
| rs10849438 | 12 | 6412036 | G/T | 0.12 | 0.113 | 1.04E-10 |
| rs10951878 | 7 | 46926695 | C/T | 0.49 | 0.058 | 1.10E-08 |
| rs11692435 | 2 | 98275354 | G/A | 0.9 | 0.113 | 1.22E-08 |
| rs12522693 | 5 | 130195731 | G/A | 0.85 | 0.01 | 3.51E-01 |
| rs12603526 | 17 | 800593 | C/T | 0.02 | 0.049 | 1.54E-01 |
| rs12635946 | 3 | 112916918 | C/T | 0.62 | 0.077 | 1.02E-11 |
| rs12979278 | 19 | 49218602 | T/C | 0.53 | 0.068 | 6.11E-10 |
| rs13020391 | 2 | 219184436 | C/T | 0.63 | 0.086 | 2.47E-13 |
| rs1321310 | 6 | 36623124 | C/T | 0.24 | 0.086 | 5.80E-11 |
| rs1330889 | 13 | 78,609,615 | C/T | 0.87 | 0.104 | 6.50E-10 |
| rs1412834 | 9 | 22110131 | T/C | 0.5 | 0.077 | 4.13E-14 |
| rs16878812 | 6 | 35569562 | A/G | 0.89 | 0.104 | 1.67E-09 |
| rs16892766 | 8 | 117630683 | C/A | 0.09 | 0.231 | 1.21E-35 |
| rs16969681 | 15 | 32993111 | T/C | 0.09 | 0.095 | 2.85E-24 |
| rs17035289 | 4 | 106048291 | T/C | 0.83 | 0.095 | 2.73E-10 |
| rs1741640 | 20 | 60932414 | C/T | 0.77 | 0.148 | 2.09E-28 |
| rs17816465 | 15 | 33156386 | A/G | 0.2 | 0.104 | 8.36E-15 |
| rs17836917 | 17 | 32047282 | G/A | 0.98 | 0.068 | 2.47E-01 |
| rs186722897 | 4 | 163374639 | T/A | 0.05 | 0.095 | 2.01E-03 |
| rs2070699 | 6 | 12292772 | T/G | 0.48 | 0.068 | 3.88E-09 |
| rs2179593 | 20 | 42660286 | A/C | 0.72 | 0.068 | 4.62E-09 |
| rs2193352 | 10 | 101346609 | G/A | 0.19 | 0.104 | 1.74E-15 |
| rs2295444 | 20 | 33173883 | C/T | 0.50 | 0.02 | 2.57E-02 |
| rs3087967 | 11 | 111156836 | T/C | 0.30 | 0.14 | 6.01E-33 |
| rs3131043 | 6 | 30758466 | G/A | 0.43 | 0.068 | 2.67E-08 |
| rs35446936 | 3 | 169486508 | G/A | 0.70 | 0.068 | 6.12E-08 |
| rs3787089 | 20 | 62316630 | C/T | 0.32 | 0.068 | 5.80E-09 |
| rs3801081 | 7 | 47511161 | G/A | 0.68 | 0.077 | 2.00E-11 |
| rs3987 | 4 | 118759055 | G/A | 0.38 | 0.02 | 9.74E-02 |
| rs4450168 | 11 | 10286755 | C/A | 0.17 | 0.095 | 1.24E-08 |
| rs45597035 | 13 | 73649152 | A/G | 0.64 | 0.077 | 2.16E-10 |
| rs4776316 | 15 | 67007813 | A/G | 0.73 | 0.077 | 1.11E-08 |
| rs4811050 | 20 | 48980670 | A/G | 0.18 | 0.086 | 4.07E-09 |
| rs4944940 | 11 | 74415252 | G/A | 0.96 | 0.039 | 3.21E-17 |
| rs6065668 | 20 | 42532821 | C/T | 0.74 | 0.02 | 1.79E-01 |
| rs6066825 | 20 | 47340117 | A/G | 0.65 | 0.095 | 3.82E-17 |
| rs6085661 | 20 | 6693128 | T/C | 0.39 | 0.086 | 2.95E-15 |
| rs6091213 | 20 | 49384745 | C/T | 0.26 | 0.077 | 5.68E-10 |
| rs61336918 | 16 | 80007266 | A/T | 0.29 | 0.086 | 2.04E-12 |
| rs61776719 | 1 | 38461319 | C/A | 0.45 | 0.068 | 2.19E-10 |
| rs6928864 | 6 | 105966894 | C/A | 0.91 | 0.122 | 1.37E-08 |
| rs6933790 | 6 | 41672769 | T/C | 0.83 | 0.095 | 3.65E-10 |
| rs72647484 | 1 | 22587728 | T/C | 0.91 | 0.122 | 2.20E-10 |
| rs73975588 | 17 | 816741 | A/C | 0.87 | 0.095 | 8.71E-09 |
| rs7398375 | 12 | 57540848 | C/G | 0.72 | 0.086 | 3.91E-10 |
| rs7495132 | 15 | 91172901 | T/C | 0.12 | 0.104 | 7.92E-10 |
| rs75686861 | 4 | 145621328 | A/G | 0.1 | 0.113 | 1.76E-09 |
| rs77776598 | 5 | 1240998 | C/T | 0.06 | 0.049 | 2.84E-10 |
| rs7894531 | 10 | 8734761 | G/A | 0.69 | 0.122 | 4.49E-24 |
| rs7993934 | 13 | 111074915 | T/C | 0.65 | 0.077 | 3.03E-11 |
| rs812481 | 3 | 66442435 | G/C | 0.52 | 0.01 | 4.38E-01 |
| rs847208 | 16 | 86254051 | A/C | 0.63 | 0.01 | 5.26E-01 |
| rs899244 | 16 | 86700030 | T/C | 0.21 | 0.086 | 1.13E-10 |
| rs9797885 | 19 | 41873001 | G/A | 0.71 | 0.077 | 2.77E-10 |
| rs9831861 | 3 | 53088285 | G/T | 0.59 | 0.068 | 4.17E-10 |
| SNP: single nucleotide polymorphism, EAF: effect allele frequency, SE: standard error, GWAS: genome-wide association study, PRS: polygenic risk score. | | | | | | |

**Supplementary Table 3: Associations of early-life factors with EOCRC risk.**

|  |  | **Incident + prevalent EOCRC** | | | | | | |  |  | **Incident EOCRC** | | | | | | |
| --- | --- | --- | --- | --- | --- | --- | --- | --- | --- | --- | --- | --- | --- | --- | --- | --- | --- |
|  | **Cases** | **Model 1** | | |  | **Model 2** | | |  | **Cases** | **Model 1** | | |  | **Model 2** | | |
|  |  | **OR** | **(95%CI)** | **P** |  | **OR** | **(95%CI)** | **P** |  |  | **OR** | **(95%CI)** | **P** |  | **OR** | **(95%CI)** | **P** |
| **Birth weight** |  |  |  |  |  |  |  |  |  |  |  |  |  |  |  |  |  |
| 2500 - 3999 g | 270 | 1.00 | (Ref) |  |  | 1.00 | (Ref) |  |  | 59 | 1.00 | (Ref) |  |  | 1.00 | (Ref) |  |
| < 2500 g | 35 | 1.07 | (0.75, 1.52) | 0.712 |  | 1.06 | (0.74, 1.51) | 0.752 |  | 4 | 0.70 | (0.25, 1.93) | 0.492 |  | 0.70 | (0.25, 1.90) | 0.471 |
| ≥ 4000 g | 52 | 1.07 | (0.80, 1.45) | 0.639 |  | 1.07 | (0.80, 1.45) | 0.640 |  | 7 | 0.75 | (0.34, 1.66) | 0.482 |  | 0.75 | (0.34, 1.65) | 0.470 |
| 1-SD increment | 357 | 1.02 | (0.92, 1.14) | 0.655 |  | 1.03 | (0.92, 1.14) | 0.617 |  | 70 | 0.97 | (0.75, 1.25) | 0.826 |  | 0.97 | (0.76, 1.26) | 0.845 |
| **Part of a multiple birth** |  |  |  |  |  |  |  |  |  |  |  |  |  |  |  |  |  |
| No | 573 | 1.00 | (Ref) |  |  | 1.00 | (Ref) |  |  | 98 | 1.00 | (Ref) |  |  | 1.00 | (Ref) |  |
| Yes | 13 | 0.97 | (0.56, 1.68) | 0.908 |  | 0.97 | (0.56, 1.68) | 0.904 |  | 3 | 1.32 | (0.42, 4.18) | 0.633 |  | 1.31 | (0.41, 4.14) | 0.647 |
| **Breastfed as baby** |  |  |  |  |  |  |  |  |  |  |  |  |  |  |  |  |  |
| Yes | 147 | 1.00 | (Ref) |  |  | 1.00 | (Ref) |  |  | 36 | 1.00 | (Ref) |  |  | 1.00 | (Ref) |  |
| No | 316 | 1.00 | (0.82, 1.23) | 0.973 |  | 1.00 | (0.82, 1.22) | 0.996 |  | 43 | 0.94 | (0.60, 1.47) | 0.780 |  | 0.95 | (0.60, 1.50) | 0.827 |
| **Comparative body size to peers at age 10 y** | | | | |  |  |  |  |  |  |  |  |  |  |  |  |  |
| About average | 103 | 1.00 | (Ref) |  |  | 1.00 | (Ref) |  |  | 12 | 1.00 | (Ref) |  |  | 1.00 | (Ref) |  |
| Thinner | 298 | 0.97 | (0.81, 1.16) | 0.731 |  | 0.97 | (0.81, 1.16) | 0.727 |  | 53 | 1.00 | (0.65, 1.54) | 0.996 |  | 1.00 | (0.65, 1.54) | 0.996 |
| Plumper | 187 | 1.09 | (0.87, 1.36) | 0.451 |  | 1.09 | (0.87, 1.36) | 0.475 |  | 35 | 0.66 | (0.35, 1.23) | 0.191 |  | 0.65 | (0.35, 1.22) | 0.183 |
| **Age at menarche** |  |  |  |  |  |  |  |  |  |  |  |  |  |  |  |  |  |
| 12 y or younger | 110 | 1.00 | (Ref) |  |  | 1.00 | (Ref) |  |  | 18 | 1.00 | (Ref) |  |  | 1.00 | (Ref) |  |
| 13 -14 y | 130 | 1.02 | (0.79, 1.31) | 0.888 |  | 1.02 | (0.79, 1.32) | 0.874 |  | 28 | 1.22 | (0.67, 2.20) | 0.513 |  | 1.22 | (0.67, 2.20) | 0.516 |
| Older than 14y | 44 | 0.93 | (0.66, 1.32) | 0.682 |  | 0.92 | (0.65, 1.31) | 0.654 |  | 8 | 0.97 | (0.42, 2.22) | 0.936 |  | 0.97 | (0.42, 2.24) | 0.946 |
| Continuous (years) | 284 | 1.00 | (0.99, 1.02) | 0.569 |  | 1.00 | (0.93, 1.08) | 0.960 |  | 54 | 1.01 | (1.00, 1.02) | 0.149 |  | 1.04 | (0.87, 1.23) | 0.683 |
| **Menstrual cycle** |  |  |  |  |  |  |  |  |  |  |  |  |  |  |  |  |  |
| Regular cycle | 63 | 1.00 | (Ref) |  |  | 1.00 | (Ref) |  |  | 40 | 1.00 | (Ref) |  |  | 1.00 | (Ref) |  |
| Irregular cycle | 12 | 0.90 | (0.48, 1.68) | 0.741 |  | 0.89 | (0.48, 1.67) | 0.724 |  | 3 | 0.47 | (0.14, 1.52) | 0.206 |  | 0.46 | (0.14, 1.51) | 0.202 |
| **Relative age of first facial hair** |  |  |  |  |  |  |  |  |  |  |  |  |  |  |  |  |  |
| About average | 232 | 1.00 | (Ref) |  |  | 1.00 | (Ref) |  |  | 38 | 1.00 | (Ref) |  |  | 1.00 | (Ref) |  |
| Younger than average | 25 | 1.17 | (0.77, 1.77) | 0.458 |  | 1.17 | (0.77, 1.77) | 0.460 |  | 5 | 1.12 | (0.44, 2.85) | 0.810 |  | 1.12 | (0.44, 2.84) | 0.817 |
| Older than average | 33 | 0.79 | (0.55, 1.14) | 0.212 |  | 0.79 | (0.55, 1.15) | 0.218 |  | 3 | 0.37 | (0.11, 1.19) | 0.095 |  | 0.37 | (0.11, 1.19) | 0.095 |
| **Relative age voice broke** |  |  |  |  |  |  |  |  |  |  |  |  |  |  |  |  |  |
| About average | 248 | 1.00 | (Ref) |  |  | 1.00 | (Ref) |  |  | 39 | 1.00 | (Ref) |  |  | 1.00 | (Ref) |  |
| Younger than average | 15 | 1.13 | (0.67, 1.91) | 0.643 |  | 1.13 | (0.67, 1.90) | 0.651 |  | 2 | 0.75 | (0.18, 3.10) | 0.690 |  | 0.74 | (0.18, 3.07) | 0.678 |
| Older than average | 14 | 0.76 | (0.44, 1.30) | 0.315 |  | 0.75 | (0.44, 1.29) | 0.294 |  | 2 | 0.53 | (0.13, 2.18) | 0.376 |  | 0.51 | (0.12, 2.12) | 0.355 |
| **Childhood sunburn occasions (times)** | 449 | 0.99 | (0.96, 1.02) | 0.368 |  | 0.99 | (0.96, 1.02) | 0.375 |  | 76 | 1.00 | (0.96, 1.04) | 0.991 |  | 1.00 | (0.95, 1.05) | 0.966 |
| Model 1: adjusted for age, sex, PRS and the top 5 principal components. Models for age at menarche (women only) , age of first facial hair (men only) and age of voice break (men only) were not adjusted for sex. Models for genetic risk were not adjusted for PRS.  Model 2: adjusted for age, sex, education(college/non-college), family history of CRC(yes/no), type 1 diabetes(yes/no), PRS and the top 5 principal components. Models for age at menarche (women only) , age of first facial hair (men only) and age of voice break (men only) were not adjusted for sex. Models for genetic risk were not adjusted for PRS. | | | | | | | | | | | | | | | | | |

**Supplementary Table 4: Association between early-life factors and EOCRC risk by polygenic risk score.**

|  |  | **Incident + prevalent cases** | | | | | | | | | | | | | | | |  |
| --- | --- | --- | --- | --- | --- | --- | --- | --- | --- | --- | --- | --- | --- | --- | --- | --- | --- | --- |
|  |  | **Low genetic risk** | | | | | | |  |  | **High genetic risk** | | | | | | |  |
|  | **Cases** | **Model 1** | | |  | **Model 2** | | |  | **Cases** | **Model 1** | | |  | **Model 2** | | | **P _interaction_** |
|  |  | **OR** | **(95%CI)** | **P** |  | **OR** | **(95%CI)** | **P** |  |  | **OR** | **(95%CI)** | **P** |  | **OR** | **(95%CI)** | **P** |  |
| **Birth weight** |  |  |  |  |  |  |  |  |  |  |  |  |  |  |  |  |  |  |
| 2500 - 3999 g | 77 | 1.00 | (Ref) |  |  | 1.00 | (Ref) |  |  | 193 | 1.00 | (Ref) |  |  | 1.00 | (Ref) |  |  |
| < 2500 g | 14 | 1.45 | (0.82, 2.57) | 0.205 |  | 1.44 | (0.81, 2.55) | 0.213 |  | 21 | 0.91 | (0.58, 1.43) | 0.687 |  | 0.90 | (0.57, 1.42) | 0.654 |  |
| ≥ 4000 g | 18 | 1.27 | (0.76, 2.13) | 0.366 |  | 1.26 | (0.75, 2.12) | 0.376 |  | 34 | 1.00 | (0.69, 1.44) | 0.979 |  | 1.00 | (0.69, 1.44) | 0.990 |  |
| 1-SD increment | 109 | 0.97 | (0.81, 1.17) | 0.773 |  | 0.97 | (0.81, 1.17) | 0.774 |  | 248 | 1.05 | (0.92, 1.19) | 0.462 |  | 1.05 | (0.93, 1.20) | 0.428 | 0.597 |
| **Part of a multiple birth** |  |  |  |  |  |  |  |  |  |  |  |  |  |  |  |  |  |  |
| No | 193 | 1.00 | (Ref) |  |  | 1.00 | (Ref) |  |  | 380 | 1.00 | (Ref) |  |  | 1.00 | (Ref) |  |  |
| Yes | 1 | 0.22 | (0.03, 1.57) | 0.131 |  | 0.22 | (0.03, 1.55) | 0.128 |  | 12 | 1.35 | (0.76, 2.41) | 0.304 |  | 1.36 | (0.76, 2.41) | 0.299 | 0.027 |
| **Breastfed as baby** |  |  |  |  |  |  |  |  |  |  |  |  |  |  |  |  |  |  |
| Yes | 101 | 1.00 | (Ref) |  |  | 1.00 | (Ref) |  |  | 215 | 1.00 | (Ref) |  |  | 1.00 | (Ref) |  |  |
| No | 45 | 0.99 | (0.69, 1.42) | 0.956 |  | 0.99 | (0.69, 1.42) | 0.941 |  | 102 | 1.01 | (0.79, 1.29) | 0.938 |  | 1.01 | (0.79, 1.28) | 0.963 | 0.861 |
| **Comparative body size to peers at age 10 y** | | | | |  |  |  |  |  |  |  |  |  |  |  |  |  |  |
| About average | 94 | 1.00 | (Ref) |  |  | 1.00 | (Ref) |  |  | 204 | 1.00 | (Ref) |  |  | 1.00 | (Ref) |  |  |
| Thinner | 62 | 1.02 | (0.74, 1.40) | 0.910 |  | 1.02 | (0.74, 1.41) | 0.904 |  | 125 | 0.95 | (0.76, 1.18) | 0.622 |  | 0.94 | (0.76, 1.18) | 0.611 |  |
| Plumper | 37 | 1.26 | (0.86, 1.85) | 0.233 |  | 1.25 | (0.86, 1.84) | 0.244 |  | 66 | 1.01 | (0.77, 1.34) | 0.928 |  | 1.01 | (0.76, 1.33) | 0.952 | 0.700 |
| **Age at menarche** |  |  |  |  |  |  |  |  |  |  |  |  |  |  |  |  |  |  |
| 12 y or younger | 40 | 1.00 | (Ref) |  |  | 1.00 | (Ref) |  |  | 70 | 1.00 | (Ref) |  |  | 1.00 | (Ref) |  |  |
| 13 -14 y | 38 | 0.82 | (0.52, 1.27) | 0.370 |  | 0.82 | (0.53, 1.28) | 0.384 |  | 92 | 1.14 | (0.83, 1.55) | 0.415 |  | 1.14 | (0.83, 1.55) | 0.419 |  |
| older than 14y | 11 | 0.64 | (0.33, 1.25) | 0.191 |  | 0.64 | (0.33, 1.24) | 0.187 |  | 33 | 1.10 | (0.72, 1.66) | 0.666 |  | 1.09 | (0.72, 1.65) | 0.695 |  |
| continuous (years) | 89 | 1.01 | (0.99, 1.03) | 0.183 |  | 0.92 | (0.81, 1.05) | 0.206 |  | 195 | 1.00 | (0.98, 1.02) | 0.914 |  | 1.04 | (0.96, 1.14) | 0.350 | 0.396 |
| **Menstrual cycle** |  |  |  |  |  |  |  |  |  |  |  |  |  |  |  |  |  |  |
| Regular cycle | 20 | 1.00 | (Ref) |  |  | 1.00 | (Ref) |  |  | 43 | 1.00 | (Ref) |  |  | 1.00 | (Ref) |  |  |
| Irregular cycle | 2 | 0.45 | (0.10, 1.96) | 0.290 |  | 0.45 | (0.10, 1.97) | 0.293 |  | 10 | 1.13 | (0.56, 2.27) | 0.739 |  | 1.11 | (0.55, 2.24) | 0.771 | 0.276 |
| **Relative age of first facial hair** |  |  |  |  |  |  |  |  |  |  |  |  |  |  |  |  |  |  |
| About average | 78 | 1.00 | (Ref) |  |  | 1.00 | (Ref) |  |  | 154 | 1.00 | (Ref) |  |  | 1.00 | (Ref) |  |  |
| Younger than average | 8 | 1.14 | (0.55, 2.37) | 0.717 |  | 1.15 | (0.56, 2.40) | 0.700 |  | 17 | 1.18 | (0.71, 1.95) | 0.518 |  | 1.17 | (0.71, 1.94) | 0.535 |  |
| Older than average | 13 | 0.94 | (0.52, 1.70) | 0.838 |  | 0.95 | (0.53, 1.71) | 0.862 |  | 20 | 0.72 | (0.45, 1.14) | 0.161 |  | 0.72 | (0.45, 1.15) | 0.168 | 0.809 |
| **Relative age voice broke** |  |  |  |  |  |  |  |  |  |  |  |  |  |  |  |  |  |  |
| About average | 89 | 1.00 | (Ref) |  |  | 1.00 | (Ref) |  |  | 159 | 1.00 | (Ref) |  |  | 1.00 | (Ref) |  |  |
| Younger than average | 3 | 0.63 | (0.20, 2.00) | 0.437 |  | 0.63 | (0.20, 2.01) | 0.439 |  | 12 | 1.42 | (0.79, 2.55) | 0.247 |  | 1.40 | (0.78, 2.53) | 0.258 |  |
| Older than average | 3 | 0.47 | (0.15, 1.48) | 0.197 |  | 0.47 | (0.15, 1.48) | 0.196 |  | 11 | 0.92 | (0.50, 1.70) | 0.787 |  | 0.90 | (0.49, 1.66) | 0.739 | 0.234 |
| **Childhood sunburn occasions (times)** | 146 | 0.98 | (0.92, 1.04) | 0.451 |  | 0.98 | (0.92, 1.04) | 0.514 |  | 303 | 0.99 | (0.95, 1.03) | 0.578 |  | 0.99 | (0.95, 1.03) | 0.545 | 0.692 |
| Model 1: adjusted for age, sex and the top 5 principal components. Models for age at menarche (women only) , age of first facial hair (men only) and age of voice break (men only) were not adjusted for sex.  Model 2: adjusted for age, sex, education(college/non-college), family history of CRC(yes/no), type 1 diabetes(yes/no) and the top 5 principal components. Models for age at menarche (women only) , age of first facial hair (men only) and age of voice break (men only) were not adjusted for sex. | | | | | | | | | | | | | | | | | | |

**Supplementary Table 5: Association between early-life factors and risk of early-onset colorectal neoplasm by gender, family history, and anatomic sites^1^.**

|  | **Female** | | |  | **Male** | | |  | **Without family history of CRC** | | |  | **With family history of CRC** | | |  | **Proximal** | | |  | **Distal** | | |  | **Rectum** | | |
| --- | --- | --- | --- | --- | --- | --- | --- | --- | --- | --- | --- | --- | --- | --- | --- | --- | --- | --- | --- | --- | --- | --- | --- | --- | --- | --- | --- |
|  | **OR** | **(95%CI)** | **P** |  | **OR** | **(95%CI)** | **P** |  | **OR** | **(95%CI)** | **P** |  | **OR** | **(95%CI)** | **P** |  | **OR** | **(95%CI)** | **P** |  | **OR** | **(95%CI)** | **P** |  | **OR** | **(95%CI)** | **P** |
| **EOCRC** |  |  |  |  |  |  |  |  |  |  |  |  |  |  |  |  |  |  |  |  |  |  |  |  |  |  |  |
| LRAU during early life | | | |  |  |  |  |  |  |  |  |  |  |  |  |  |  |  |  |  |  |  |  |  |  |  |  |
| No | 1.00 | (Ref) |  |  | 1.00 | (Ref) |  |  | 1.00 | (Ref) |  |  | 1.00 | (Ref) |  |  | 1.00 | (Ref) |  |  | 1.00 | (Ref) |  |  | 1.00 | (Ref) |  |
| Yes | 1.60 | (1.00, 2.53) | 0.047 |  | 1.26 | (0.62, 2.54) | 0.525 |  | 1.32 | (0.86, 2.04) | 0.208 |  | 2.34 | (1.01, 5.43) | 0.047 |  | 1.52 | (0.69, 3.36) | 0.303 |  | 1.32 | (0.68, 2.56) | 0.417 |  | 1.72 | (0.81, 3.67) | 0.161 |
| **EOCRA** |  |  |  |  |  |  |  |  |  |  |  |  |  |  |  |  |  |  |  |  |  |  |  |  |  |  |  |
| LRAU during early life | | | |  |  |  |  |  |  |  |  |  |  |  |  |  |  |  |  |  |  |  |  |  |  |  |  |
| No | 1.00 | (Ref) |  |  | 1.00 | (Ref) |  |  | 1.00 | (Ref) |  |  | 1.00 | (Ref) |  |  | 1.00 | (Ref) |  |  | 1.00 | (Ref) |  |  | 1.00 | (Ref) |  |
| Yes | 1.30 | (1.03, 1.64) | 0.028 |  | 1.54 | (1.17, 2.03) | 0.002 |  | 1.46 | (1.20, 1.77) | <0.001 |  | 1.09 | (0.68, 1.74) | 0.719 |  | 1.92 | (1.21, 3.05) | 0.006 |  | 1.43 | (0.95, 2.15) | 0.086 |  | 1.46 | (1.05, 2.04) | 0.025 |

^1^Adjusted for age, sex, education (college/non-college), family history of CRC (yes/no), type 1 diabetes (yes/no) and the top 5 principal components. Models for age at menarche (women only) , age of first facial hair (men only) and age of voice break (men only) were not adjusted for sex.

**Supplementary Table 6: Risk of Early-Onset Neoplasm by joint categorization for genetic risk and LRAU during early life.**

| PRS level | Cases | **Model 1** | | |  | **Model 2** | | |
| --- | --- | --- | --- | --- | --- | --- | --- | --- |
|  |  | OR (95% CI) | | P value |  | OR (95% CI) | | P value |
| **EOCRC (cases, n = 165)** |  |  |  |  |  |  |  |  |
| Low genetic risk |  |  |  |  |  |  |  |  |
| No LRAU during early life | 44 | 1.00 | ( Ref ) |  |  | 1.00 | ( Ref ) |  |
| LRAU during early life | 9 | 1.17 | (0.57, 2.41) | 0.665 |  | 1.17 | (0.57, 2.40) | 0.665 |
| High genetic risk |  |  |  |  |  |  |  |  |
| No LRAU during early life | 87 | 1.98 | (1.38, 2.85) | <0.001 |  | 1.98 | (1.38, 2.84) | <0.001 |
| LRAU during early life | 25 | 3.23 | (1.97, 5.31) | <0.001 |  | 3.23 | (1.97, 5.31) | <0.001 |
| **EOCRA (cases, n = 719)** |  |  |  |  |  |  |  |  |
| Low genetic risk |  |  |  |  |  |  |  |  |
| No LRAU during early life | 204 | 1.00 | ( Ref ) |  |  | 1.00 | ( Ref ) |  |
| LRAU during early life | 60 | 1.41 | (1.05, 1.89) | 0.020 |  | 1.41 | (1.05, 1.88) | 0.022 |
| High genetic risk |  |  |  |  |  |  |  |  |
| No LRAU during early life | 353 | 1.71 | (1.44, 2.04) | <0.001 |  | 1.67 | (1.41, 1.99) | <0.001 |
| LRAU during early life | 102 | 2.39 | (1.88, 3.04) | <0.001 |  | 2.34 | (1.83, 2.98) | <0.001 |
| Model 1: adjusted for age, sex and the top 5 principal components.  Model 2: adjusted for age, sex, education(college/non-college), family history of CRC(yes/no),  type 1 diabetes(yes/no) and the top 5 principal components. | | | | | | | | |

**Supplementary Table 7: Associations of LRAU during early life and polygenic risk score with early-onset colorectal neoplasm risk using cox regression.**

|  |  |  | **Incident + prevalent cases** | | | | | | |  |  | **Incident cases** | | | | | | |
| --- | --- | --- | --- | --- | --- | --- | --- | --- | --- | --- | --- | --- | --- | --- | --- | --- | --- | --- |
|  |  | **Cases** | **Model 1** | | |  | **Model 2** | | |  | **Cases** | **Model 1** | | |  | **Model 2** | | |
|  |  |  | **HR** | **(95%CI)** | **P** |  | **HR** | **(95%CI)** | **P** |  |  | **HR** | **(95%CI)** | **P** |  | **HR** | **(95%CI)** | **P** |
| **EOCRC** | **LRAU during early life** |  |  |  |  |  |  |  |  |  |  |  |  |  |  |  |  |  |
|  | No | 131 | 1.00 | (Ref) |  |  | 1.00 | (Ref) |  |  | 15 | 1.00 | (Ref) |  |  | 1.00 | (Ref) |  |
|  | Yes | 34 | 1.48 | (1.01, 2.17) | 0.044 |  | 1.48 | (1.01, 2.16) | 0.046 |  | 7 | 1.88 | (0.76, 4.65) | 0.173 |  | 1.86 | (0.75, 4.61) | 0.182 |
|  | **Polygenetic risk score (PRS)** |  |  |  |  |  |  |  |  |  |  |  |  |  |  |  |  |  |
|  | Low genetic risk | 200 | 1.00 | (Ref) |  |  | 1.00 | (Ref) |  |  | 29 | 1.00 | (Ref) |  |  | 1.00 | (Ref) |  |
|  | High genetic risk | 394 | 2.03 | (1.71, 2.41) | < 0.001 |  | 1.99 | (1.67, 2.36) | < 0.001 |  | 72 | 2.51 | (1.62, 3.88) | < 0.001 |  | 2.47 | (1.60, 3.83) | < 0.001 |
|  | Continuous | 594 | 2.33 | (2.01, 2.69) | < 0.001 |  | 2.27 | (1.97, 2.62) | < 0.001 |  | 101 | 2.53 | (1.79, 3.59) | < 0.001 |  | 2.50 | (1.76, 3.54) | < 0.001 |
| **EOCRA** | **LRAU during early life** |  |  |  |  |  |  |  |  |  |  |  |  |  |  |  |  |  |
|  | No | 557 | 1.00 | (Ref) |  |  | 1.00 | (Ref) |  |  | 195 | 1.00 | (Ref) |  |  | 1.00 | (Ref) |  |
|  | Yes | 162 | 1.40 | (1.17, 1.67) | < 0.001 |  | 1.40 | (1.17, 1.67) | < 0.001 |  | 52 | 1.16 | (0.85, 1.58) | 0.349 |  | 1.15 | (0.84, 1.56) | 0.389 |
|  | **Polygenetic risk score (PRS)** |  |  |  |  |  |  |  |  |  |  |  |  |  |  |  |  |  |
|  | Low genetic risk | 920 | 1.00 | (Ref) |  |  | 1.00 | (Ref) |  |  | 336 | 1.00 | (Ref) |  |  | 1.00 | (Ref) |  |
|  | High genetic risk | 1430 | 1.52 | (1.39, 1.65) | < 0.001 |  | 1.48 | (1.36, 1.61) | < 0.001 |  | 495 | 1.43 | (1.24, 1.64) | < 0.001 |  | 1.41 | (1.23, 1.62) | < 0.001 |
|  | Continuous | 2350 | 1.62 | (1.50, 1.74) | < 0.001 |  | 1.57 | (1.46, 1.69) | < 0.001 |  | 831 | 1.58 | (1.40, 1.79) | < 0.001 |  | 1.56 | (1.38, 1.76) | < 0.001 |
| Model 1: adjusted for age, sex, PRS and the top 5 principal components. Models for genetic risk were not adjusted for PRS.  Model 2: adjusted for age, sex, education(college/non-college), family history of CRC(yes/no), type 1 diabetes(yes/no), PRS and the top 5 principal components.  Models for genetic risk were not adjusted for PRS. | | | | | | | | | | | | | | | | | | |

**Supplementary Table 8: Association between LRAU during early life and risk of early-onset colorectal neoplasm by polygenic risk score using cox regression.**

|  |  |  | **Incident + prevalent cases** | | | | | | | | | | | | | | | |  |
| --- | --- | --- | --- | --- | --- | --- | --- | --- | --- | --- | --- | --- | --- | --- | --- | --- | --- | --- | --- |
|  |  |  | **Low genetic risk** | | | | | | |  |  | **High genetic risk** | | | | | | |  |
|  |  | **Cases** | **Model 1** | | |  | **Model 2** | | |  | **Cases** | **Model 1** | | |  | **Model 2** | | | **P _interaction_** |
|  |  |  | **HR** | **(95%CI)** | **P** |  | **HR** | **(95%CI)** | **P** |  |  | **HR** | **(95%CI)** | **P** |  | **HR** | **(95%CI)** | **P** |  |
| **EOCRC** | LRAU during early life | | | | |  |  |  |  |  |  |  |  |  |  |  |  |  |  |
|  | No | 44 | 1.00 | (Ref) |  |  | 1.00 | (Ref) |  |  | 87 | 1.00 | (Ref) |  |  | 1.00 | (Ref) |  |  |
|  | Yes | 9 | 1.08 | (0.52, 2.23) | 0.840 |  | 1.05 | (0.51, 2.18) | 0.886 |  | 25 | 1.71 | (1.09, 2.69) | 0.020 |  | 1.72 | (1.09, 2.70) | 0.020 | 0.420 |
| **EOCRA** | LRAU during early life | | | | |  |  |  |  |  |  |  |  |  |  |  |  |  |  |
|  | No | 204 | 1.00 | (Ref) |  |  | 1.00 | (Ref) |  |  | 353 | 1.00 | (Ref) |  |  | 1.00 | (Ref) |  |  |
|  | Yes | 60 | 1.43 | (1.07, 1.91) | 0.016 |  | 1.42 | (1.06, 1.90) | 0.019 |  | 102 | 1.38 | (1.11, 1.73) | 0.004 |  | 1.38 | (1.11, 1.73) | 0.004 | 0.666 |
| Model 1: adjusted for age, sex and the top 5 principal components. Models for genetic risk were not adjusted for PRS.  Model 2: adjusted for age, sex, education(college/non-college), family history of CRC(yes/no), type 1 diabetes(yes/no) and the top 5 principal components. | | | | | | | | | | | | | | | | | | | |

**Supplementary Table 9: Associations of LRAU and polygenic risk score with EOCRC risk after using age 55 as the cutoff to define EOCRC.**

|  |  | **Incident + prevalent cases** | | | | | | |  |  | **Incident cases** | | | | | | |
| --- | --- | --- | --- | --- | --- | --- | --- | --- | --- | --- | --- | --- | --- | --- | --- | --- | --- |
|  | **Cases** | **Model 1** | | |  | **Model 2** | | |  | **Cases** | **Model 1** | | |  | **Model 2** | | |
|  |  | **OR** | **(95%CI)** | **P** |  | **OR** | **(95%CI)** | **P** |  |  | **OR** | **(95%CI)** | **P** |  | **OR** | **(95%CI)** | **P** |
| **LRAU during early life** | | | | |  |  |  |  |  |  |  |  |  |  |  |  |  |
| No | 283 | 1.00 | (Ref) |  |  | 1.00 | (Ref) |  |  | 62 | 1.00 | (Ref) |  |  | 1.00 | (Ref) |  |
| Yes | 64 | 1.30 | (0.98, 1.71) | 0.064 |  | 1.29 | (0.98, 1.70) | 0.070 |  | 23 | 1.56 | (0.96, 2.54) | 0.070 |  | 1.51 | (0.93, 2.46) | 0.092 |
| **Polygenetic risk score (PRS)** |  |  |  |  |  |  |  |  |  |  |  |  |  |  |  |  |  |
| Low genetic risk | 105 | 1.00 | (Ref) |  |  | 1.00 | (Ref) |  |  | 27 | 1.00 | (Ref) |  |  | 1.00 | (Ref) |  |
| High genetic risk | 242 | 2.31 | (1.84, 2.91) | <0.001 |  | 2.27 | (1.80, 2.85) | <0.001 |  | 58 | 2.11 | (1.34, 3.34) | 0.001 |  | 2.06 | (1.30, 3.25) | 0.002 |
| Continuous | 347 | 2.67 | (2.21, 3.22) | <0.001 |  | 2.62 | (2.17, 3.16) | <0.001 |  | 85 | 2.55 | (1.74, 3.72) | <0.001 |  | 2.46 | (1.68, 3.61) | <0.001 |
| Model 1: adjusted for age, sex, PRS and the top 5 principal components. Models for genetic risk were not adjusted for PRS.  Model 2: adjusted for age, sex, education(college/non-college), family history of CRC(yes/no), type 1 diabetes(yes/no), PRS and the top 5 principal components. Models for genetic risk were not adjusted for PRS. | | | | | | | | | | | | | | | | | |

**Supplementary Table 10: Association between LRAU and EOCRC risk by polygenic risk score after using age 55 as the cutoff to define EOCRC.**

|  |  | **Incident + prevalent cases** | | | | | | | | | | | | | | | |  |
| --- | --- | --- | --- | --- | --- | --- | --- | --- | --- | --- | --- | --- | --- | --- | --- | --- | --- | --- |
|  |  | **Low genetic risk** | | | | | | |  |  | **High genetic risk** | | | | | | |  |
|  | **Cases** | **Model 1** | | |  | **Model 2** | | |  | **Cases** | **Model 1** | | |  | **Model 2** | | | **P _interaction_** |
|  |  | **OR** | **(95%CI)** | **P** |  | **OR** | **(95%CI)** | **P** |  |  | **OR** | **(95%CI)** | **P** |  | **OR** | **(95%CI)** | **P** |  |
| **LRAU during early life** | | | | |  |  |  |  |  |  |  |  |  |  |  |  |  |  |
| No | 90 | 1.00 | (Ref) |  |  | 1.00 | (Ref) |  |  | 193 | 1.00 | (Ref) |  |  | 1.00 | (Ref) |  |  |
| Yes | 15 | 0.90 | (0.52, 1.56) | 0.696 |  | 0.89 | (0.51, 1.55) | 0.680 |  | 49 | 1.50 | (1.09, 2.07) | 0.013 |  | 1.49 | (1.08, 2.05) | 0.015 | 0.197 |
| Model 1: adjusted for age, sex and the top 5 principal components.  Model 2: adjusted for age, sex, education(college/non-college), family history of CRC(yes/no), type 1 diabetes(yes/no) and the top 5 principal components. | | | | | | | | | | | | | | | | | | |

**Supplementary table 11: Gene-antibiotic interaction estimates for the risk of Early-onset Colorectal Neoplasm.**

|  | **Gene: FUT2** | | | | |  | **Gene-antibiotic interaction effect** (SNP× Early-life antibiotic use(yes/no)) | | | | |
| --- | --- | --- | --- | --- | --- | --- | --- | --- | --- | --- | --- |
|  | **Model 1** | |  | **Model 2** | |  | **Model 1** | |  | **Model 2** | |
|  | OR (95% CI) | P value |  | OR (95% CI) | P value |  | Beta | P _interaction_ |  | Beta | P _interaction_ |
| **EOCRC (cases, n = 165)** | | |  |  |  |  |  |  |  |  |  |
| rs35866622 | 1.08 (0.87, 1.34) | 0.498 |  | 1.08 (0.87, 1.34) | 0.492 |  | -0.001 | 0.999 |  | -0.002 | 0.994 |
| rs601338 | 1.10 (0.89, 1.37) | 0.386 |  | 1.10 (0.88, 1.36) | 0.396 |  | 0.069 | 0.799 |  | 0.069 | 0.801 |
| rs281377 | 1.23 (0.99, 1.54) | 0.066 |  | 1.23 (0.99, 1.54) | 0.068 |  | -0.005 | 0.985 |  | -0.004 | 0.990 |
| **EOCRA (cases, n = 719)** | | |  |  |  |  |  |  |  |  |  |
| rs35866622 | 1.02 (0.92, 1.13) | 0.694 |  | 1.02 (0.92, 1.13) | 0.691 |  | -0.126 | 0.319 |  | -0.129 | 0.305 |
| rs601338 | 1.04 (0.93, 1.15) | 0.495 |  | 1.04 (0.93, 1.15) | 0.535 |  | -0.118 | 0.351 |  | -0.128 | 0.311 |
| rs281377 | 1.05 (0.94, 1.16) | 0.391 |  | 1.05 (0.94, 1.16) | 0.380 |  | 0.220 | 0.083 |  | 0.216 | 0.089 |
| Model 1: adjusted for age, sex and the top 5 principal components.  Model 2: adjusted for age, sex, education(college/non-college), family history of CRC (yes/no),  type 1 diabetes(yes/no) and the top 5 principal components. | | | | | | | | | | | |

**
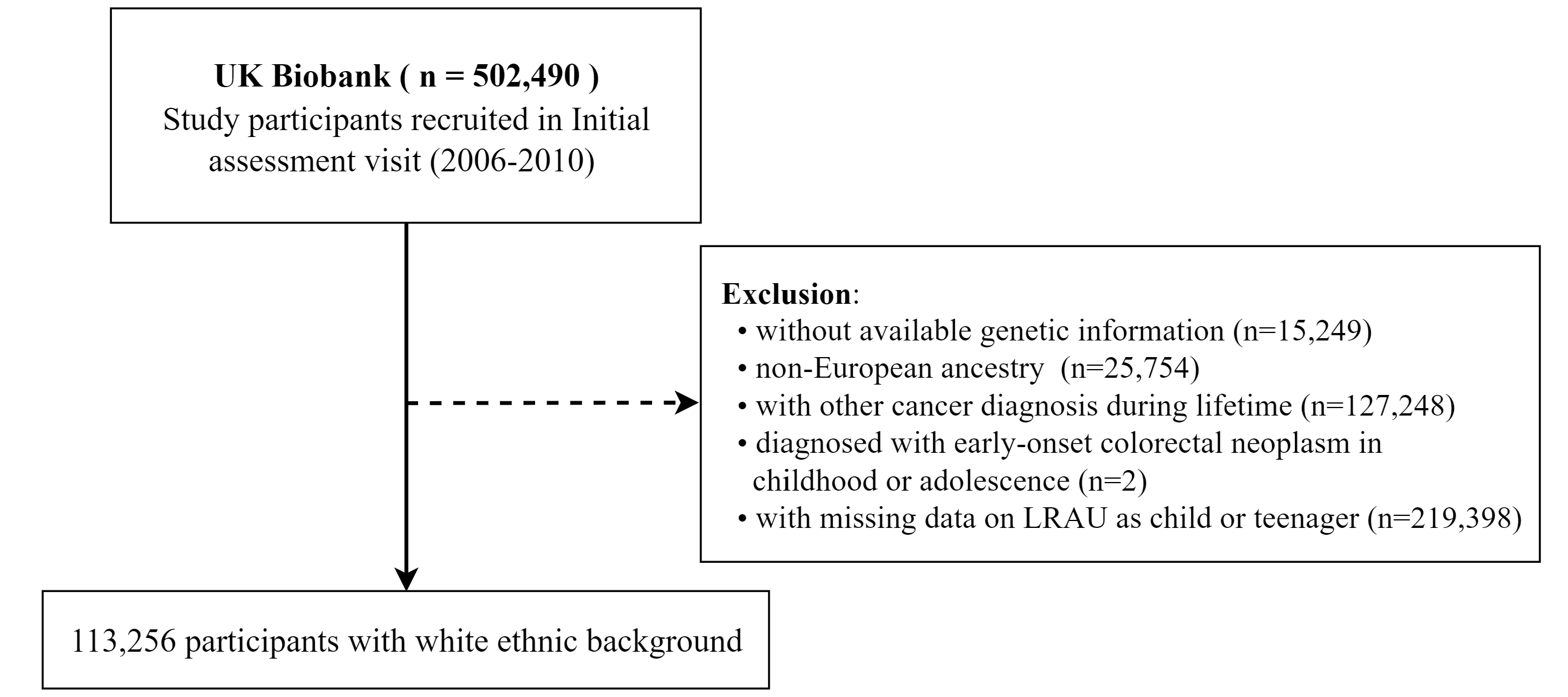
**

**Supplementary Figure 1. Flowchart of study population selection.**
